# Interpretable machine learning for predicting the response duration to Sintilimab plus chemotherapy in patients with advanced gastric or gastroesophageal junction cancer

**DOI:** 10.3389/fimmu.2024.1407632

**Published:** 2024-05-22

**Authors:** Dan-qi Wang, Wen-huan Xu, Xiao-wei Cheng, Lei Hua, Xiao-song Ge, Li Liu, Xiang Gao

**Affiliations:** ^1^ Big Data Center, Affiliated Hospital of Jiangnan University, Wuxi, China; ^2^ Department of Oncology, Affiliated Hospital of Jiangnan University, Wuxi, China

**Keywords:** Sintilimab, immunotherapy, progression-free survival, machine learning, interpretability

## Abstract

**Background:**

Sintilimab plus chemotherapy has proven effective as a combination immunotherapy for patients with advanced gastric and gastroesophageal junction adenocarcinoma (GC/GEJC). A multi-center study conducted in China revealed a median progression-free survival (PFS) of 7.1 months. However, the prediction of response duration to this immunotherapy has not been thoroughly investigated. Additionally, the potential of baseline laboratory features in predicting PFS remains largely unexplored. Therefore, we developed an interpretable machine learning (ML) framework, iPFS-SC, aimed at predicting PFS using baseline (pre-treatment) laboratory features and providing interpretations of the predictions.

**Materials and methods:**

A cohort of 146 patients with advanced GC/GEJC, along with their baseline laboratory features, was included in the iPFS-SC framework. Through a forward feature selection process, predictive baseline features were identified, and four ML algorithms were developed to categorize PFS duration based on a threshold of 7.1 months. Furthermore, we employed explainable artificial intelligence (XAI) methodologies to elucidate the relationship between features and model predictions.

**Results:**

The findings demonstrated that LightGBM achieved an accuracy of 0.70 in predicting PFS for advanced GC/GEJC patients. Furthermore, an F1-score of 0.77 was attained for identifying patients with PFS durations shorter than 7.1 months. Through the feature selection process, we identified 11 predictive features. Additionally, our framework facilitated the discovery of relationships between laboratory features and PFS.

**Conclusion:**

A ML-based framework was developed to predict Sintilimab plus chemotherapy response duration with high accuracy. The suggested predictive features are easily accessible through routine laboratory tests. Furthermore, XAI techniques offer comprehensive explanations, both at the global and individual level, regarding PFS predictions. This framework enables patients to better understand their treatment plans, while clinicians can customize therapeutic approaches based on the explanations provided by the model.

## 1 Introduction

Gastric and gastroesophageal junction adenocarcinoma (GC/GEJC) is the fifth most common cancer worldwide and approximately 44% of patients are diagnosed in China (1). The median overall survival is approximately one year for advanced GC/GEJC. Recently, immune checkpoint blockade therapy targeting programmed death 1 ligand (PD-L1) has shown efficacy in HER2-negative GC/GEJC (1).

Sintilimab is a recombinant, fully human IgG4 anti-PD-1 monoclonal antibody, which is the earliest approved anti-PD-1 monoclonal antibody for gastric cancer in China (2). The multicenter ORIENT-16 randomized clinical trial conducted across 62 hospitals in China demonstrated that the addition of Sintilimab to chemotherapy significantly enhanced overall patient-specific outcomes, including overall survival (OS) and median progression-free survival (PFS), in all 650 previously untreated patients with advanced GC/GEJC (3). Specifically, the study found that patients treated with Sintilimab, who had a combined positive score (CPS) of 5 or more, exhibited a median PFS of 7.1 months compared to those receiving placebo and chemotherapy (3). However, it’s noteworthy that CPS testing can only be conducted in hospitals primarily located in tertiary settings, and relevant tests are still unavailable in rural hospitals. Additionally, the current detection platforms for CPS show inconsistencies, leading to significant deviations across different platforms.

As an alternative to CPS, PFS and OS have been utilized to predict survival outcomes in gastric cancer (GC) through methods like Kaplan-Meier analysis and Cox proportional hazards models (4–8). Among these studies, Ozveren et al. (6) identified an association between the inflammatory prognostic index (IPS) score (derived from C-reactive protein (CRP), neutrophil-to-lymphocyte ratio (NLR), and serum albumin) and the risk of disease progression, highlighting the potential utility of clinical laboratory tests in predicting tumor response.

Machine learning (ML) methods offer a valuable approach to analyzing intricate datasets and revealing underlying relationships between predictors and outcomes. By employing ML techniques, it becomes feasible to capture non-linear associations among features, thereby enhancing prediction accuracy. Previous research has demonstrated the efficacy of various ML algorithms, including Logistic Regression (LR), Naïve Bayes (NB), Support Vector Machine (SVM), k-Nearest Neighbors (KNN), Decision Tree (DT), Random Forest (RF), eXtreme Gradient Boosting (XGBoost), and convolutional neural networks (CNN), in effectively predicting outcomes in GC (9–16). To date, there has been no systematic application of ML algorithms to predict patient survival following treatment with Sintilimab plus chemotherapy, and it remains uncertain whether baseline laboratory features are associated with short-term survival outcomes, such as short PFS.

To tackle these challenges, we utilized four ML algorithms within a local cohort comprising 146 patients diagnosed with advanced GC/GEJC. Our aim was to develop a novel framework for predicting treatment response, termed iPFS-SC (Interpretable machine learning models for predicting Progression-Free Survival in patients undergoing Sintilimab plus Chemotherapy). Within this framework, we evaluated the potential of baseline laboratory tests to forecast the response to Sintilimab plus chemotherapy. Considering the median PFS of 7.1 months observed with Sintilimab plus chemotherapy (3), we utilized the threshold of 7.1 months to categorize treatment outcomes. To streamline the feature set and minimize redundancy, we employed a forward feature selection method, which identified a subset of 11 baseline laboratory features relevant to our task of classifying PFS duration. These features included mean corpuscular hemoglobin (MCH) in whole blood, urinary osmolality (SG-STY), and serum creatinine (CREA), etc. In particular, the LightGBM model, in conjunction with the chosen features, emerged as the best-performing model, attaining an accuracy and weighted F1-score of 0.70 and 0.71, correspondingly, in predicting the PFS of patients with advanced GC/GEJC.

As ML is often perceived as a black box within the healthcare system, posing challenges to its reliability due to the need for clinicians to explain specific predictions to patients (17, 18). We incorporated cutting-edge explainable artificial intelligence (XAI) methods (19–22), such as SHapley Additive exPlanations (SHAP) and Diverse counterfactual explanations (DiCE), into the LightGBM model. These approaches enabled us to generate both global and individualized explanations for predictive laboratory features. The results demonstrated that LightGBM effectively captured both linear and nonlinear relationships between features and outputs, identified important thresholds of features, and established simple constraints on features for generating counterfactuals. Therefore, the proposed iPFS-SC framework not only offered high accuracy for predicting PFS but also provided explainable analysis of laboratory features. This capability is particularly valuable for individualized PFS prediction in patients with advanced GC/GEJC.

## 2 Methods and materials

### 2.1 General setup of iPFS-SC framework

**Figure 1:**
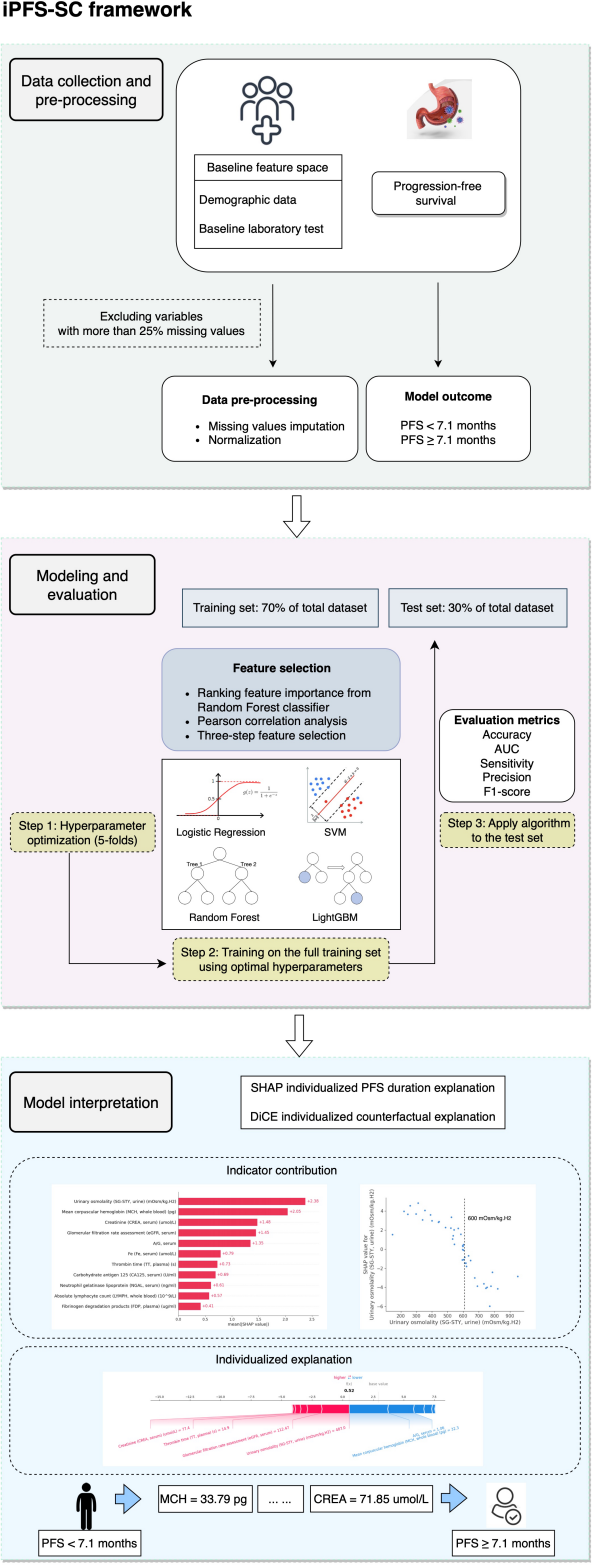
The overview of iPFS-SC framework.

**Figure 1** provides an overview of the iPFS-SC framework. Within iPFS-SC, baseline features encompassing demographic and laboratory test data from 146 patients with advanced GC/GEJC were gathered alongside their corresponding PFS durations. The aim of iPFS-SC was to utilize interpretable ML methodologies to predict patients’ PFS, with a specified threshold at 7.1 months. As part of our study, we examined 113 laboratory features, allowing for a maximum missingness threshold of 25%. Among these, 11 features were identified as predictive variables. We then employed four ML algorithms (LR, SVM, RF, and LightGBM), with hyperparameters determined via 5-fold cross-validation within the training set. Evaluation metrics including accuracy, area under the receiver operating curve (AUC), sensitivity, precision, and F1-score were calculated for each algorithm. Furthermore, model interpretation was generated through the utilization of SHAP and DiCE algorithms within the best-performing algorithm.

### 2.2 Patient enrollment

The study included patients diagnosed with advanced GC/GEJC, who underwent treatment with Sintilimab plus chemotherapy at the Affiliated Hospital of Jiangnan University from January 16, 2020, to January 26, 2024. Clinical response to Sintilimab plus chemotherapy was assessed using the Response Evaluation Criteria in Solid Tumors (RECIST) criteria. Computed tomography (CT) scans and magnetic resonance imaging (MRI) were conducted at the initial visit and during the 2-year follow-up to evaluate tumor status.

### 2.3 Data preparation

#### 2.3.1 Population characteristics

The demographic data (age, sex, and smoking status) and laboratory test (whole blood, plasma, serum, urine, gastric fluid, fecal, sputum, etc.) were initially assessed at Patients’ baseline visits. Features with missing values exceeding 25% were excluded, resulting in 113 features across five categories: demographics (n=3), whole blood (n=24), plasma (n=49), serum (n=10), and urine (n=27). The existing missing values were imputed by Missforest (Python missingpy library (version 0.2.0)), a non-parametric random forest imputation algorithm that can cope with numerical and categorical variables simultaneously (23).

#### 2.3.2 PFS outcomes

Derived from the findings of Xu et al. (3), the median PFS was 7.1 months for patients in Sintilimab plus chemotherapy treatment. Employing this PFS threshold of 7.1 months, we created outcome labels for the iPFS-SC framework. Patients who experienced a PFS duration of 7.1 months or longer were categorized as one group (n=53), while those with a PFS shorter than 7.1 months (n=93) were classified into another group.

### 2.4 Feature selection

The dataset was split into training and test sets (7:3) in a stratified manner. We utilized StandardScaler from the Python Scikit-learn library (version 1.1.2) to normalize the data. The most predictive laboratory features were then identified through a three-step selection process within the training set. Firstly, feature importance was assessed by calculating the mean decrease in impurity (MDI) using a naïve RF classifier. The MDI for a feature is determined by calculating the average reduction in impurity resulting from splitting on that feature across all nodes in an RF classifier (24). We then ranked the features in descending order based on their MDI values, with the one exhibiting the highest ranking deemed the most crucial for predicting PFS. Secondly, to address multicollinearity, pairwise Pearson correlation coefficients were computed between these features, with coefficients exceeding 0.70 subjected to further scrutiny. For the elimination process, the feature with comparatively lower MDI feature importance among the correlated features was then excluded. Lastly, the final feature corpus was constructed by iteratively incorporating the remaining features from the previous step into an RF classifier, with the cumulative AUC being evaluated at each iteration. The iterative process concluded when no further improvement in the cumulative AUC was observed.

### 2.5 iPFS-SC modeling and evaluation

Four ML algorithms were employed, including LR, SVM, RF, and LightGBM. To determine the optimal hyperparameters for each algorithm, we utilized 5-fold cross-validation within the training set via Python Optuna framework (version 3.5.0). During cross-validation, four folds were utilized for tuning hyperparameters, while one fold was reserved to assess model performance. Throughout the training process, the Tree-structured Parzen Estimator (TPE) (25) was designated as a sampler with the number of trials configured to 50. We aimed to maximize and evaluate the AUC. Subsequently, we retrained the model using the suggested hyperparameters and assessed the performance of each algorithm on unseen test data. The hyperparameter ranges for each algorithm are summarized in **Supplementary Table 1**. The best-performing model was integrated into the iPFS-SC framework.

### 2.6 Statistical analysis

The distribution of each feature was evaluated against the Kolmogorov-Smirnov test. To compare between the two groups (PFS< 7.1 months vs. PFS ≥ 7.1 months), Welch’s 2-sample t-test was employed, assuming normal distribution of continuous data. The significance level for both statistical tests was set at 0.05. Model classification performance was assessed using metrics including AUC, accuracy, recall, precision, and F1-score (the harmonic mean of recall and precision). All statistical analyses were conducted using the Python scikit-learn library (version 1.1.2) and scipy library (version 1.9.1).

### 2.7 Interpretation of iPFS-SC

Model explanations were generated by analyzing feature attribution to predict PFS within the test set. To achieve this, we utilized two methodologies. Firstly, the SHAP algorithm (21) was employed to uncover global feature attributions, with higher magnitudes indicating greater contributions to shorter PFS. Furthermore, the relationship between chosen features and model output was investigated through partial dependence plots. Additionally, the SHAP force plot was used to illustrate the influence of each variable on the final SHAP value, offering localized, sample-specific explanations. These SHAP analyses provided a thorough understanding of the reasoning behind iPFS-SC’s particular PFS predictions and supplied feature importance for individual samples.

Secondly, counterfactual explanation was conducted to produce interpretable changes in features aimed at achieving the desired model prediction, transitioning from PFS< 7.1 months to PFS ≥ 7.1 months. This methodology involved exploring “what-if” scenarios, where the goal was to determine the outcome if the selected feature was altered. Specifically, given the model’s outcome y_i_ = 0 (indicating PFS shorter than 7.1 months) for the input feature space x_i_:{x_0_, x_1_, …, x_10_}, we aimed to ascertain the outcome when the selected feature is changed to x_i_’:{x_0_’, x_1_’, …,x_10_’}. Throughout our study, the modified inputs (cases from the test set) were fed into the trained ML model. Additionally, the DiCE algorithm (22) was employed to generate a set of counterfactual explanations. These implementations and visualizations were conducted using the Python shap library (version 0.44.1) and dice-ml library (version 0.11).

## 3 Results

### 3.1 The baseline characteristics of the study population

Our study involved 146 patients who received Sintilimab plus chemotherapy. The median [IQR] age of the cohort was 68 [60, 73], with 105 [71.9%] male and 41 [28.1%] female participants. Among them, 93 patients had a PFS shorter than 7.1 months, with a median age of 67 [60, 73], comprising 67 [72.0%] male and 26 [28.0%] female individuals. Conversely, the long PFS group consisted of 53 patients, with a median age of 69 [60, 73], and comprised 38 [71.7%] male and 15 [28.3%] female participants. Statistical descriptions of the features utilized in iPFS-SC are presented in **Table 1**, with numerical features displayed as median [IQR]. The features listed were found to be normally distributed (Kolmogorov-Smirnov test, P-value< 0.0001).

**Table 1:** The statistical characteristics of laboratory features assessed as important for PFS prediction.

Feature	Full name	Clinical specimen	Missing Percentage (%)	Total (median [IQR])	PFS< 7.1 months (n=93)	PFS ≥ 7.1 months (n=53)	P-value (Welch’s 2-sample t-test)
MCH	Mean corpuscular hemoglobin (pg)	Whole blood	0.68	29.8 [26.7, 31.6]	29.3 [25.78, 31.05]	30.9 [28.8, 32.2]	0.001
LYMPH	Absolute lymphocyte count (10^9^/L)		0.68	1.2 [1.0, 1.5]	1.2 [0.98, 1.5]	1.3 [1.1, 1.7]	0.031
SG-STY	Urinary osmolality (mOsm/kg.H2)	Urine	18.49	550.0 [416.5, 664.0]	514.5 [389.5, 615.0]	620 [522, 746]	0.002
Fe	Fe ( μmol/L )	Serum	4.79	8.2 [5.2, 13.18]	7.85 [4.74, 12.74]	10.0 [6.37, 15.58]	0.012
eGFR	Glomerular filtration rate assessment		2.74	109.97 [87.57, 129.04]	101.21 [87.57, 124.66]	114.14 [90.45, 143.47]	0.021
CA125	Carbohydrate antigen 125 (U/mL)		0.68	177.1 [134.6, 215.1]	176 [141.88, 211.53]	184.4 [127.7, 222.5]	0.013
A/G	A/G		0.68	1.36 [1.19, 1.58]	1.36 [1.22, 1.60]	1.38 [1.13, 1.56]	0.016
NGAL	Neutrophil gelatinase lipoprotein (ng/mL)		6.85	128.75 [88.83, 201.55]	134.55 [94.7, 236.78]	106.4 [80.88, 154.53]	0.039
CREA	Creatinine ( μmol/L )		0.68	69.1 [56.5, 81.6]	72.15 [58.48, 81.38]	63.5 [53.9, 82.0]	0.012
TT	Thrombin time (s)	Plasma	5.48	15.95 [15.2, 17.2]	15.6 [15.0, 16.8]	16.6 [15.8, 17.6]	0.022
FDP	Fibrinogen degradation products ( μg/mL )		5.48	4.25 [2.7, 8.38]	4.3 [2.7, 6.9]	4.2 [2.1, 10.6]	0.045

### 3.2 Identification of predictive laboratory features

**Figure 2:**
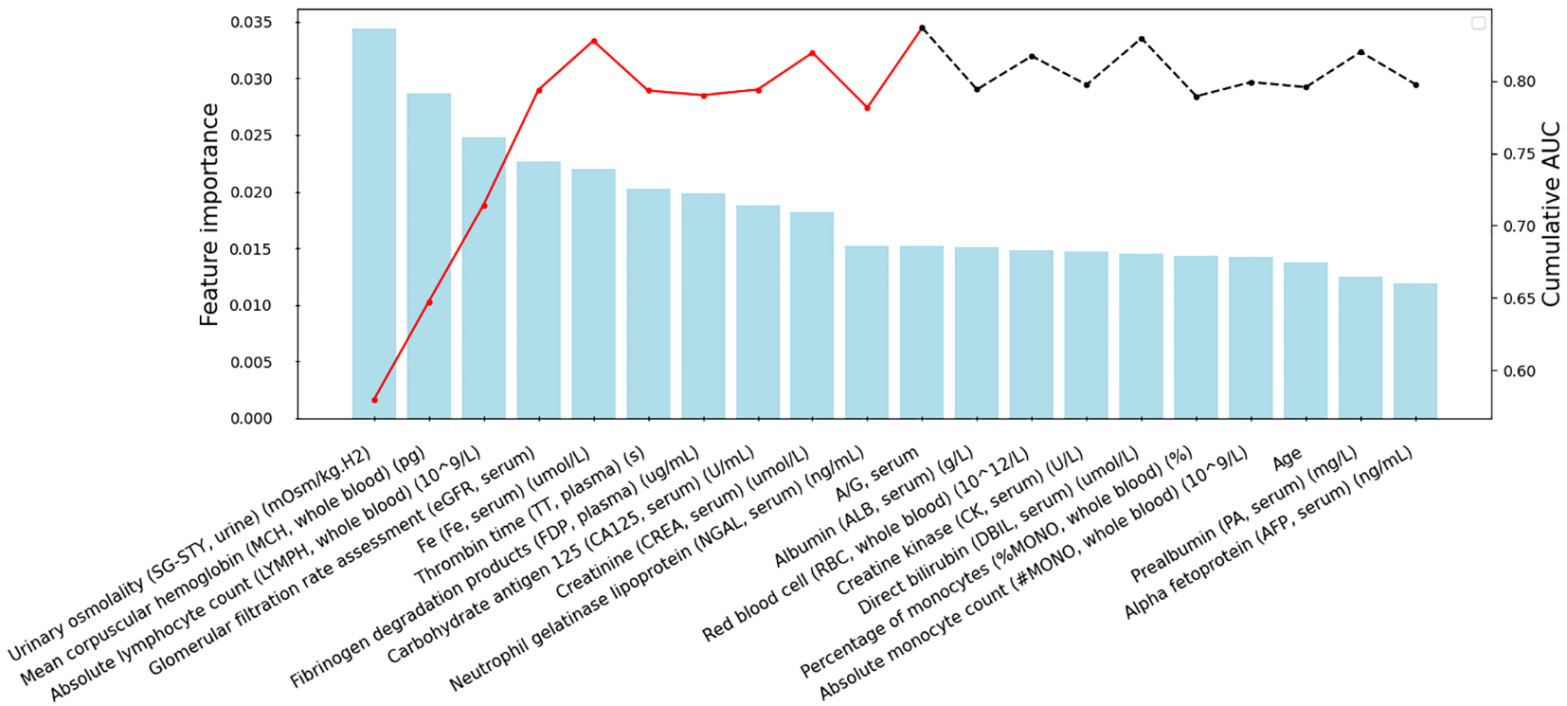
Feature selection within the iPFS-SC framework. The *x*-axis displays the selected features, arranged in descending order of importance after addressing multicollinearity. The feature importance from RF is shown on the left *y*-axis, while the cumulative AUC is presented on the right *y*-axis. The red lines indicate the final 11 features chosen for modeling.

To mitigate overfitting and minimize bias in the predictive model, we adopted a forward feature selection approach to determine the optimal number of features for iPFS-SC modeling. Initially, we identified the top 50 features based on their MDI values, ranked in descending order of importance. Subsequently, after addressing multicollinearity through Pearson correlation analysis, a total of 39 features remained for further evaluation. These features were then sequentially integrated into the RF classifier, and the cumulative AUC was computed. **Supplementary Figure 1** displays the features with Pearson correlation coefficients exceeding 0.70 and their respective MDI feature importance scores. The removed features could be found in **Supplementary Table 2**. **Figure 2** illustrates the top-ranked 20 features alongside their respective feature importance (left *y*-axis) and cumulative AUC (right *y*-axis). By sequentially incorporating 11 laboratory features, such as urinary SG-STY, whole blood MCH and whole blood LYMPH, an AUC of 0.84 was attained. Further addition of features did not yield any improvement, as indicated by the dashed line. Consequently, 11 features derived from four clinical categories (whole blood, plasma, serum, and urine) were utilized for modeling and predicting PFS.

Furthermore, the feature selection process was conducted in nine additional training/test splits. The percentages depicting the occurrence of predictive features among these splits are presented in **Supplementary Figure 2**. It is evident that, all 11 features consistently demonstrated high occurrences across various splits, highlighting the robustness of the feature selection procedure.

### 3.3 LightGBM outperformed other ML algorithms for PFS prediction

The model evaluation was conducted on assessing performance on the unseen test set. **Table 2** summarizes the evaluation metrics of various models, while **Table 3** presents the optimal hyperparameters for each algorithm. Overall, LightGBM outperformed LR, SVM, and RF, with a weighted F1-score and an accuracy of 0.71 and 0.70, respectively. Furthermore, it achieved an F1-score of 0.77 in identifying patients with short PFS. Ultimately, the LightGBM model was chosen and incorporated into the iPFS-SC framework to forecast the PFS duration of advanced GC/GEJC patients.

**Table 2:** The evaluation metrics of four ML algorithms on unseen test data.

Model	Accuracy	AUC	Category	Recall	Precision	F1-score
LR	0.68	0.65	PFS< 7.1 months	0.71	0.86	0.77
			PFS ≥ 7.1 months	0.6	0.38	0.46
			Weighted avg.	0.68	0.75	0.70
SVM	0.64	0.60	PFS< 7.1 months	0.7	0.75	0.72
			PFS ≥ 7.1 months	0.5	0.44	0.47
			Weighted avg.	0.64	0.65	0.64
RF	0.64	0.60	PFS< 7.1 months	0.68	0.82	0.74
			PFS ≥ 7.1 months	0.5	0.31	0.38
			Weighted avg.	0.64	0.71	0.66
LightGBM	0.70	0.68	PFS< 7.1 months	0.76	0.79	0.77
			PFS ≥ 7.1 months	0.6	0.56	0.58
			Weighted avg.	0.70	0.71	0.71

**Table 3:** The optimal hyperparameters of four ML algorithms.

Model	Hyperparameters
LR	C (0.14034567027876954), solver (“lbfgs”)
SVM	C (100), kernel (“rbf”)
RF	max_depth (16), max_features (“sqrt”), min_samples_leaf (5), n_estimators (10)
LightGBM	num_leaves (10), learning_rate (0.3), max_depth (8), min_child_samples (5), n_estimators (50)

### 3.4 iPFS-SC interpretation

#### 3.4.1 SHAP global and individualized PFS duration explanation

**Figure 3:**
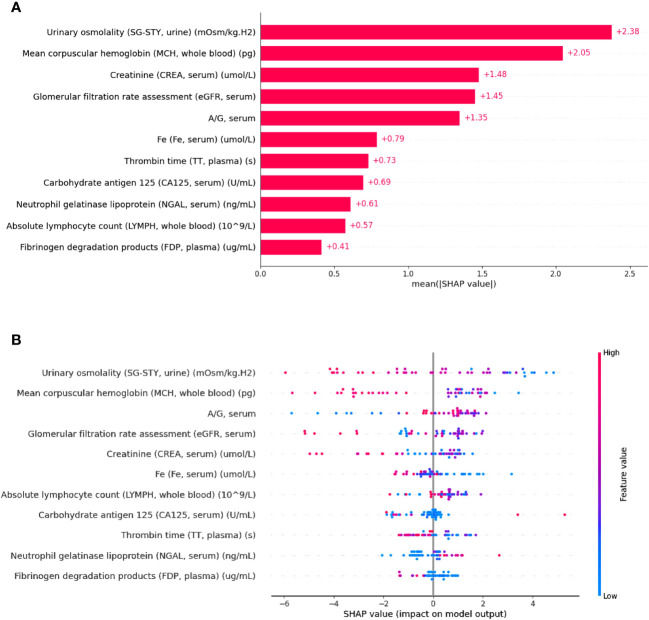
Interpretation of the LightGBM classifier using SHAP for predicting PFS. The bar plot of the mean absolute SHAP value of each feature **(A)**, the summary plot of the distribution of SHAP values and the impact of each feature on the model’s prediction **(B)**.

XAI methodologies, such as SHAP, offer model interpretability by combining feature importance visualization with model predictions. The mean absolute SHAP values depict the overall impact of each feature on the output of LightGBM within the test set. As depicted in **Figure 3A**, the top-ranking features in terms of their relative importance for predicting PFS shorter than 7.1 months were urinary SG-STY, whole blood MCH, and serum CREA, respectively. Unlike the bar plot, **Figure 3B** presents a SHAP summary plot aimed at providing a deeper understanding of the actual relationships between features and the PFS following Sintilimab plus chemotherapy. The horizontal magnitude and direction indicate the predictive strength of each feature. A positive SHAP value toward the right indicates a tendency toward short PFS (< 7.1 months), while a negative value toward the left suggests long PFS (≥ 7.1 months). Furthermore, the gradient coloring of each feature’s quantitative range, from red (high value) to blue (low value), offers insights into how the model’s prediction changes with variations in feature values. These results highlighted that urinary SG-STY, whole blood MCH, serum A/G, serum eGFR, and serum CREA exerted the greatest magnitude effects on the model’s output, indicating their critical role as predictors of combination immunotherapy response.

**Figure 4:**
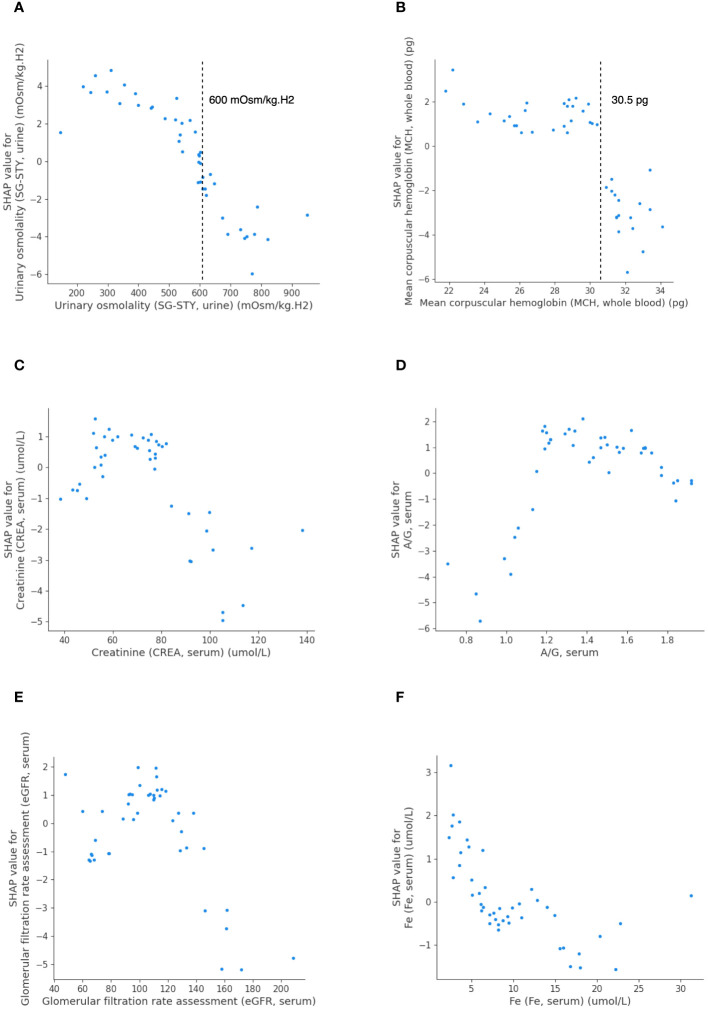
The SHAP dependence plots detailing feature values and contributions to predicting PFS in LightGBM. Urinary SG-STY **(A)**, whole blood MCH **(B)**, serum CREA **(C)**, serum A/G **(D)**, serum eGFR **(E)**, and serum Fe **(F)**.

To delve deeper into the importance of the top-ranking features for predicting PFS, we further created partial dependence plots. These plots illustrated the relationship between each feature and its effect on PFS, highlighting crucial thresholds for predicting outcomes (**Figures 4A-F**). The SHAP value on the y-axis indicated the direction of the effect on PFS (negative: PFS ≥ 7.1 months; positive: PFS< 7.1 months) when one feature changed within a certain range (x-axis). **Figure 4** suggests that LightGBM effectively captured both linear and complex relationships between the selected features and the model output. In our efforts to identify the triggering features associated with long PFS, we observed that patients with elevated levels of urinary SG-STY (> 600 mOsm/kg.H2) or whole blood MCH (> 30.5 pg) exhibited negative SHAP values (**Figures 4A, B**). More dependence plots of predictive features can be found in **Supplementary Figures 3A-E**.

**Figure 5:**
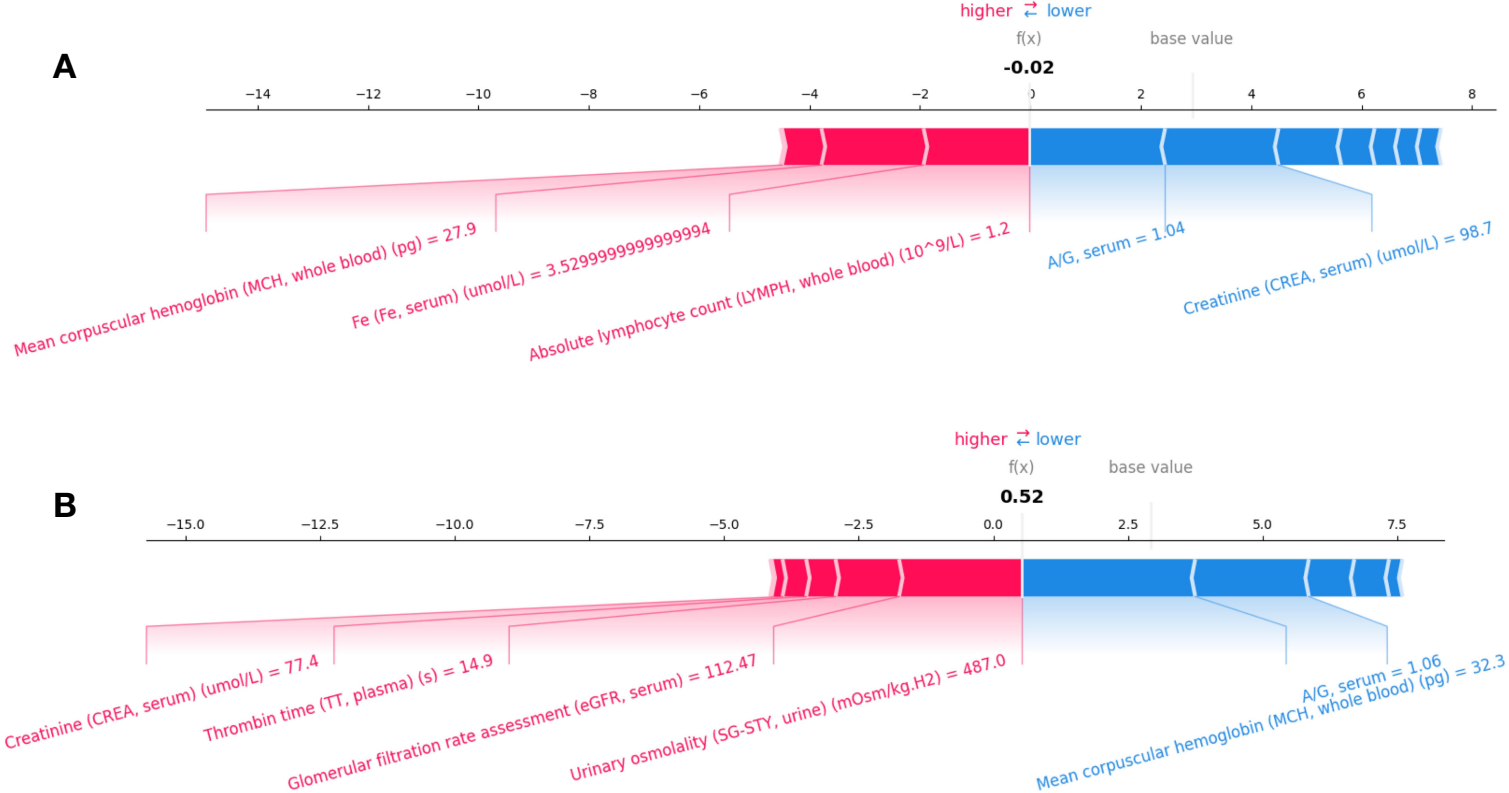
Individualized explanation of patients with PFS longer **(A)** and shorter than 7.1 months **(B)**.

**Figures 5A, B** present individualized explanations for a patient with a PFS duration longer and shorter than 7.1 months, respectively. As shown in **Figure 5A**, iPFS-SC predicted a final SHAP value of -0.02 for the individual. Feature values of serum A/G (1.04) and serum CREA (98.7 μmol/L ), indicating a longer PFS, contrast with those of whole blood MCH (27.9 pg), serum Fe (3.53 μmol/L ), and whole blood LYMPH (1.2 × 10^9^/L), which suggest a shorter PFS. In contrast to the scenario depicted in **Figure 5A**, the individual in **Figure 5B** presented a SHAP value of 0.52. The figure demonstrates that the values of serum CREA (77.4 μmol/L ), plasma TT (14.9 s), serum eGFR (112.47), and urinary SG-STY (487.0 mOsm/kg.H2) had the most significant impact on the final outcome of short PFS.

#### 3.4.2 Counterfactual explanations via DiCE

Given the explanation evidence of SHAP feature attributions to the PFS prediction, we proceeded to examine whether making minor and interpretable adjustments to a given feature could yield a contradictory outcome. To do so, we randomly chose a patient from the test set with a PFS shorter than 7.1 months and utilized the DiCE algorithm to generate counterfactual cases for LightGBM. This process aimed to identify the optimal values of predictive features that would lead to the opposite outcome (PFS ≥ 7.1 months).

**Table 4** displays the laboratory reference intervals of the selected features and one counterfactual case from test set. For the queried patient, assuming all other features remained unchanged, if the patient’s urinary SG-STY was 771.49 mOsm/kg.H2 and serum A/G was 2.04, it is anticipated that the patient would have a PFS of 7.1 months or even longer. A similar counterfactual outcome was observed when the values of whole blood MCH was 33.79 pg and serum CREA shifted to 71.85 μmol/L . Furthermore, a decline in plasma TT (14.26 s) and a significant rise in serum Fe (35.17  μmol/L ) also suggested a long PFS. More counterfactual cases were generated and displayed in **Supplementary Table 3**.

**Table 4:** DiCE counterfactual explanations of one given query case from the test set.

MCH	SG-STY	LYMPH	TT	Fe	NGAL	eGFR	FDP	CA125	CREA	A/G
Reference interval										
27-34 (pg)	600-1000 (mOsm/kg.H2)	1.1-3.2 (10^9^/L)	14-21 (s)	10.6-36.7 ( μ mol/L)	37-180 (ng/mL)	—	0-5 ( μg/mL )	0-35 (U/mL)	57-111 ( μ mol/L)	1.2-2.4
Original feature set (PFS< 7.1 months)										
32.3	487.0	1.4	14.9	8.18	179.4	112.47	10.6	41.28	77.4	1.06
Counterfactual set (PFS ≥ 7.1 months)										
–	771.49	–	–	–	–	–	–	–	–	2.04
33.79	–	–	–	–	–	–	–	–	71.85	–
–	–	–	14.26	35.17	–	–	–	–	–	–

The patient represented its original query features and the counterfactual feature set (- means no change for the given feature).

These results corroborated the findings of the SHAP interpretable analysis, confirming the relationship between quantitative features and PFS, as well as the critical thresholds that led to contrasting PFS outcomes.

## 4 Discussion

In recent years, there has been a notable evolution in immunotherapies, which are now recommended for patients diagnosed with advanced GC/GEJC (26, 27). This progress has been driven by the discovery of innovative diagnostic biomarkers, the development of drugs targeting novel molecules, and the emergence of combination therapies, such as immunotherapy combined with chemotherapy, antiangiogenic agents, anti-HER-2 antibodies, and chemotherapy (26). Despite these advancements, a portion of GC patients do not derive benefits from immunotherapy, as evidenced by the lack of improvement in their OS and PFS compared to chemotherapy alone (28).

Hence, there is a critical need to construct predictive models aimed at identifying patients who stand to benefit from immunotherapy, particularly those likely to achieve prolonged PFS. This not only aids in identifying suitable candidates for immunotherapy but also helps clinicians comprehend individual predictors, thereby enabling the adjustment of treatment strategies accordingly.

The development of iPFS-SC was grounded in the findings from the multi-center ORIENT-16 trial, which demonstrated the efficacy of Sintilimab combined with chemotherapy, resulting in a median PFS of 7.1 months for patients with advanced GC/GEJC in China (3). Consequently, our model’s outcome label was to predict whether a patient would experience a PFS shorter than 7.1 months. The increasing interest in utilizing ML solutions for developing predictive tools and uncovering characteristic features underscores the necessity of creating ML algorithms for GC research. To the best of our knowledge, ML approaches have not been employed to investigate tumor response to Sintilimab combination therapy. In line with this perspective, iPFS-SC integrated interpretable ML algorithms with baseline laboratory features, enabling accurate prediction of patients’ PFS to Sintilimab plus chemotherapy while systematically evaluating the relationship between predictive features and model outputs. Additionally, thresholds for features that generated counterfactual effects were scrutinized, allowing for a better understanding of the significance of each feature for modeling.

Based on our findings, baseline laboratory features have emerged as crucial indicators for PFS prediction. Through a three-step forward feature selection process, we pinpointed 11 predictive features from four clinical specimens (whole blood, plasma, serum, and urine). LightGBM, trained on the predictive features, surpassed LR, SVM, and RF, achieving an accuracy of 0.70 in predicting PFS of advanced GC/GEJC patients, and an F1-score of 0.77 for identifying patients with a PFS shorter than 7.1 months. During SHAP interpretable analysis, we unearthed non-linear relationships between top-ranking features and PFS, for example, whole blood MCH, serum CREA and serum A/G.

Prior research has correlated elevated MCH levels with improved survival in hepatocellular carcinoma patients, indicating its significance in cancer progression (29, 30). Our study identified that higher whole blood MCH levels (> 30.5 pg) were negatively associated with short PFS. The trigger point and the correlation identified in the SHAP analysis was corroborated by DiCE, demonstrating that elevated whole blood MCH level could lead to an opposite PFS outcome. Urinary SG-STY, incorporating ions, glucose, and urea levels, provided a reliable indicator of urine dilution (31). Despite its rare evaluation in GC studies, iPFS-SC revealed a linear relationship between urinary SG-STY and PFS, identifying a threshold of 600 mOsm/kg.H2 indicative of a PFS duration equal to or longer than 7.1 months. In summary, the predictive features identified within the iPFS-SC framework included not only well-established biomarkers in cancer progression but also less-investigated clinical features.

We acknowledge the limitations when interpreting our findings. Firstly, in addition to the cohort receiving Sintilimab plus chemotherapy, a control group of advanced GC/GEJC patients who have not undergone immunotherapy can be included in future studies. This would comprehensively identify individual patients who may benefit from Sintilimab plus chemotherapy based on their distinct clinical characteristics. Secondly, we employed XAI methodologies, including SHAP global feature importance, individualized model explanation and DiCE counterfactual effects, to elucidate the intricate relationships among predictive features and the PFS outcome. It’s important to note that the thresholds generated for features and counterfactual cases may exhibit slight variations depending on the dataset utilized. Additionally, our study was conducted using data from a single center, and the cohort size was relatively small. A multi-center study recruiting heterogeneous patient cohorts could be conducted to assess the model’s performance on a large scale.

## 5 Conclusion

In conclusion, we were the first to discover predictive features from baseline laboratory test for patients with advanced GC/GEJC receiving Sintilimab plus chemotherapy. The iPFS-SC framework was developed through the feature selection, ML-based PFS prediction, and model interpretation, enabling personalized immunotherapy. Our findings demonstrated the framework’s ability to predict PFS in patients with advanced GC/GEJC. Leveraging XAI methodologies, we revealed the contribution of features to the model output and identified thresholds for certain features to generate contrasting PFS outcomes. With a feature corpus comprising 11 laboratory features alongside the developed model, we could effectively evaluate and interpret the PFS duration of advanced GC/GEJC patients to Sintilimab plus chemotherapy.

## Data availability statement

The raw data supporting the conclusions of this article will be made available by the authors, without undue reservation.

## Ethics statement

The studies involving humans were approved by The ethics committee/institutional review board of the Affiliated Hospital of Jiangnan University. The studies were conducted in accordance with the local legislation and institutional requirements. The participants provided their written informed consent to participate in this study.

## Author contributions

DW: Writing – review & editing, Writing – original draft, Visualization, Methodology, Data curation. WX: Writing – original draft, Resources, Investigation. XC: Writing – original draft, Resources, Investigation. LH: Writing – review & editing, Investigation, Data curation. XG: Writing – review & editing, Resources, Investigation. LL: Writing – review & editing, Supervision, Project administration, Funding acquisition, Conceptualization. XG: Writing – original draft, Supervision, Resources, Project administration, Investigation, Funding acquisition, Conceptualization.

## Glossary

**Table:**

GC-GEJC	Advanced gastric and gastroesophageal junction adenocarcinoma
PFS	Progression-free survival
ML	Machine learning
iPFS-SC	Interpretable machine learning models for predicting Progression-Free Survival in patients undergoing Sintilimab plus Chemotherapy
PD-1	Programmed death 1 ligand
OS	Overall survival
CPS	Combined positive score
GC	Gastric cancer
IPS	Inflammatory prognostic index
CRP	C-reactive protein
NLR	Neutrophil-to-lymphocyte
LR	Logistic Regression
NB	Naïve Bayes
SVM	Support Vector Machine
KNN	k-Nearest Neighbors
DT	Decision Tree
RF	Random Forest
XGBoost	eXtreme Gradient Boosting
CNN	Convolutional neural network
TPE	Tree-structured Parzen Estimator
MCH	Mean corpuscular hemoglobin
SG-STY	Urinary osmolality
LYMPH	Percentage of lymphocytes
XAI	Explainable artificial intelligence
SHAP	SHapley Additive exPlanations
DiCE	Diverse Counterfactual Explanations
RECIST	Response Evaluation Criteria in Solid Tumors
CT	Computed tomography
MRI	Magnetic resonance imaging
MDI	Mean decrease in impurity
NGAL	Neutrophil gelatinase lipoprotein
CREA	Creatinine
TT	Thrombin time
eGFR	Glomerular filtration rate assessment
CA125	Carbohydrate antigen 125
FDP	Fibrinogen degradation products
