# Supplementary material for: Interpretable machine learning for predicting the response duration to Sintilimab plus chemotherapy in patients with advanced gastric or gastroesophageal junction cancer
Source: Front Immunol. 2024 May 22;15:1407632. doi: 10.3389/fimmu.2024.1407632 (PMC11150638; doi:10.3389/fimmu.2024.1407632)
Supplement: Supplementary file 1 [file DataSheet_1.pdf]

frontiers

Supplementary Material

Interpretable machine learning for predicting the response duration to Sintilimab Plus Chemotherapy in patients with gastric cancer

Dan-qi Wang[1], Wen-huan Xu[2], Xiao-wei Cheng[2], Lei Hua[1], Xiao-song Ge[2], Li Liu[1*], Xiang Gao[2*]

[1]Big Data Center, Affiliated Hospital of Jiangnan University, Wuxi, China

[2]Department of Oncology, Affiliated Hospital of Jiangnan University, Wuxi, China

*** Correspondence:**

Li Liu
9862016027@jiangnan.edu.cn

Xiang Gao
13606189128@139.com

1. Supplementary Figures and Tables

1.1. Supplementary Tables

Supplementary Table 1.

Overview of hyperparameters tuning in each algorithm.

**Model**	**Hyperparameter range**
LR	C (Loguniform (1e-4, 10000)),<br>solver (“newton-cg”, “lbfgs”, “liblinear”)
SVM	C (100, 1000),<br>kernel (“rbf”, “linear”, “sigmoid”, “poly”)
RF	max_depth (3, 16),<br>max_features (“log2”, “sqrt”),<br>min_samples_leaf (1, 3, 5, 7, 9, 11, 13, 15, 17, 19),<br>n_estimators (5, 10, 20, 30, 50)

LightGBM	num_leaves (2, 5, 10, 15, 30),<br>learning_rate (0.01, 0.1, 0.2, 0.3),<br>max_depth (2, 8),<br>min_child_samples (1, 5, 10),<br>n_estimators (10, 20, 50)

Supplementary Table 2.

The removed features based on the results obtained from Pearson correlation analysis and MDI feature importance.

**Feature names**	**Abbreviations**
Mean red blood cell volume (whole blood) (fL)	MCV
Urinay conductivity (urine) (mS/cm)	Cond.
Total protein (serum) (g/L)	TP
D-dimer test (plasma) (mg/L)	D-Di
Cystatin C (serum) (mg/L)	Cysc
Retinol binding protein (serum) (mg/L)	RBP
Indirect bilirubin (serum) (umol/L)	IBIL
Total bilirubin (serum) (umol/L)	TBIL
Glycocholic acid (serum) (mg/L)	CG
Total platelet count (whole blood) (10^9/L)	PLT
Hematocrit (whole blood) (%)	HCT

Supplementary Table 3.

DiCE counterfactual explanations of given query cases from the test set.

MCH	SG-STY	LYMPH	TT	Fe	NGAL	eGFR	FDP	CA125	CREA	A/G
**Reference interval**										
27-34 (pg)	600-1000 (mOsm/kg.H2)	1.1-3.2 (10^9/L)	14-21 (s)	10.6-36.7 (μmol/L)	37-180 (ng/mL)	---	0-5 (μg/mL)	0-35 (U/mL)	57-111 (μmol/L)	1.2-2.4
**a. Original feature set 1 (PFS < 7.1 months)**										
21.8	601	1.2	15.2	2.77	96.1	146.2	3.9	6.43	55.0	1.83
**Counterfactual set 1 (PFS ≥ 7.1 months)**										
-	-	-	-	-	-		-	-	93.29	2.91
-	-	2.34	-	-	-	-	-	-	107.39	-
31.19	-	-	-	17.90	-	-	-	-	-	-
**b. Original feature set 2 (PFS < 7.1 months)**										
22.2	244.0	0.6	15.0	7.84	120.61	66.09	9.0	27.8	117.2	1.55
**Counterfactual set 2 (PFS ≥ 7.1 months)**										
32.01	-	-	16.56	-	-	-	-	-	-	-
-	716.48	1.75	-	-	-	-	-	-	-	-
28.09	-	-	-	-	-	-	-	-	-	1.87
**c. Original feature set 3 (PFS < 7.1 months)**										
25.1	541.72	1.3	15.9	6.4	117.7	118.37	1.5	12.60	53.1	1.62
**Counterfactual set 3 (PFS ≥ 7.1 months)**										
-	805.00	-	-	-	-	-	-	-	80.81	-
29.06	930.75	-	-	-	-	-	-	-	-	-
-	837.05	-	-	-	-	-	-	-	105.11	-

Supplementary Material

1.2. Supplementary Figures

A

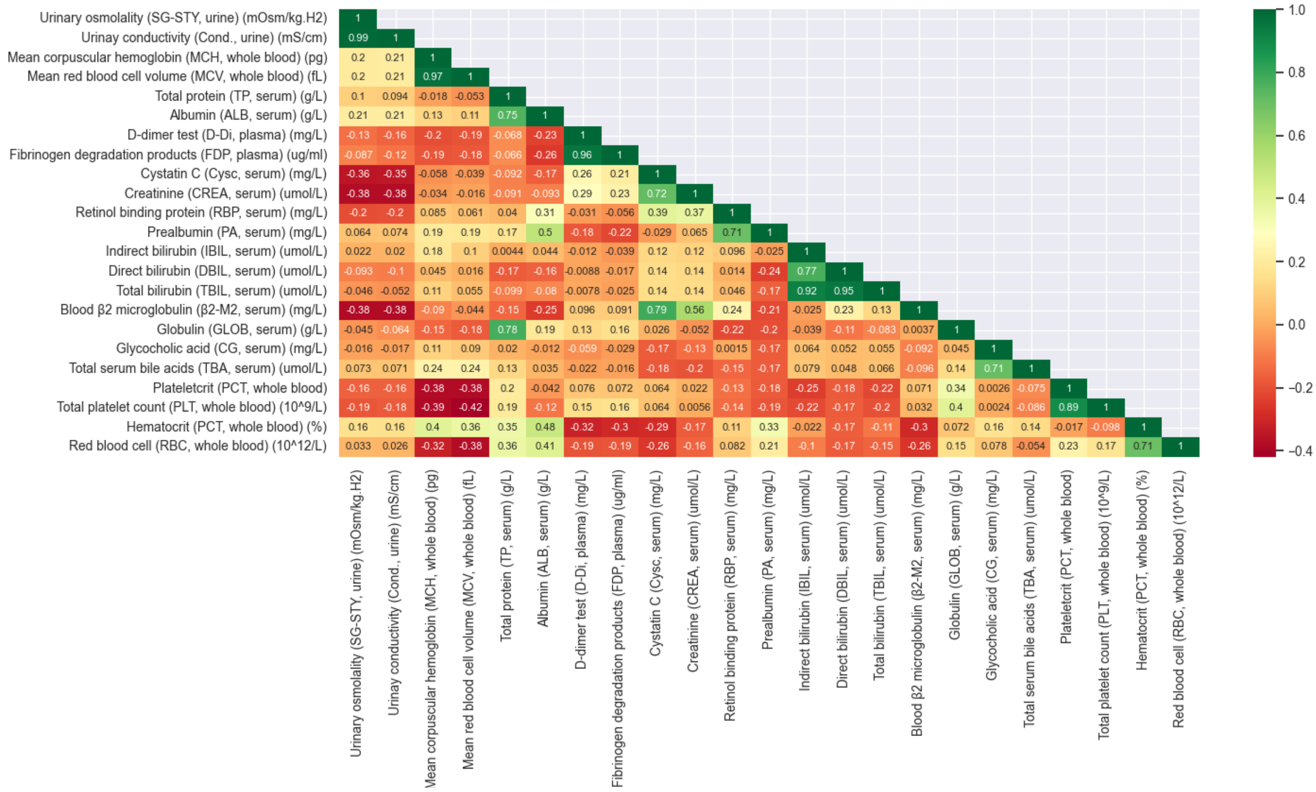

B

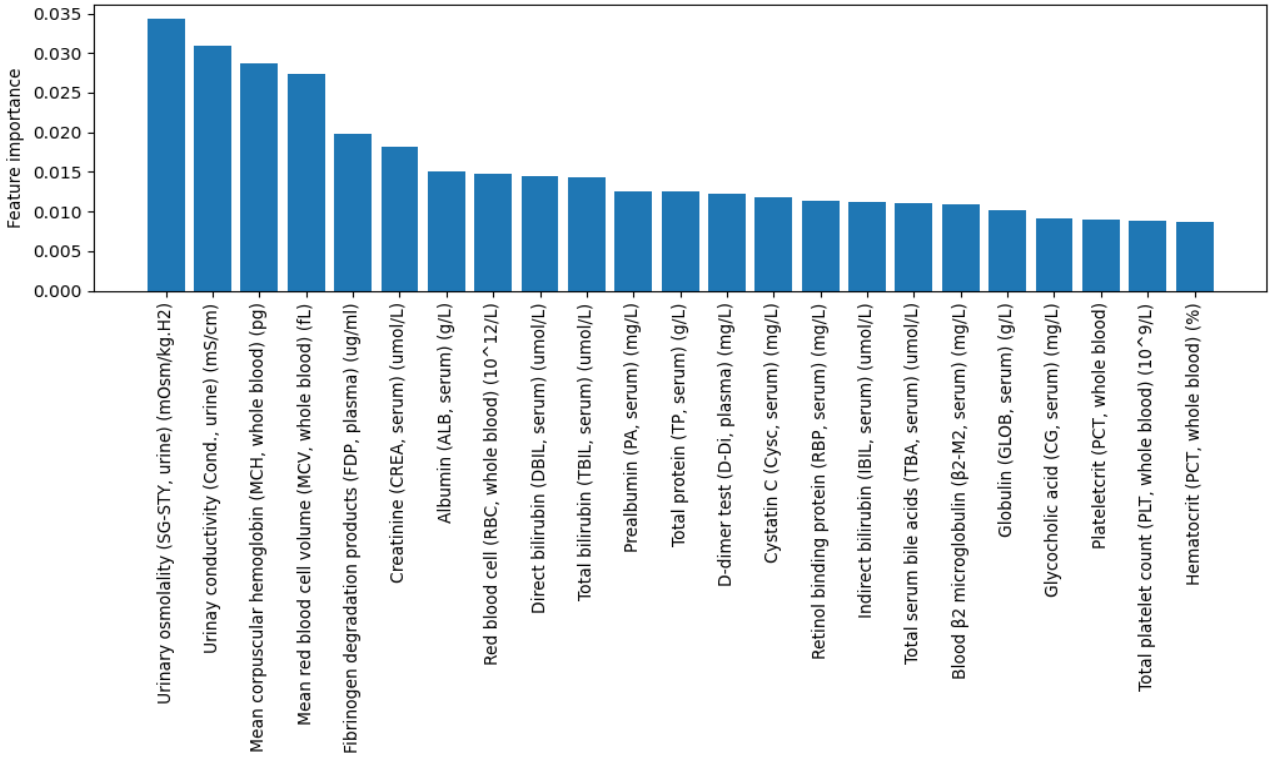

Supplementary Figure 1. The correlated features (with Pearson correlation > 0.70) from the top-ranking features (A). MDI feature importance of correlated features (B).

Supplementary Material

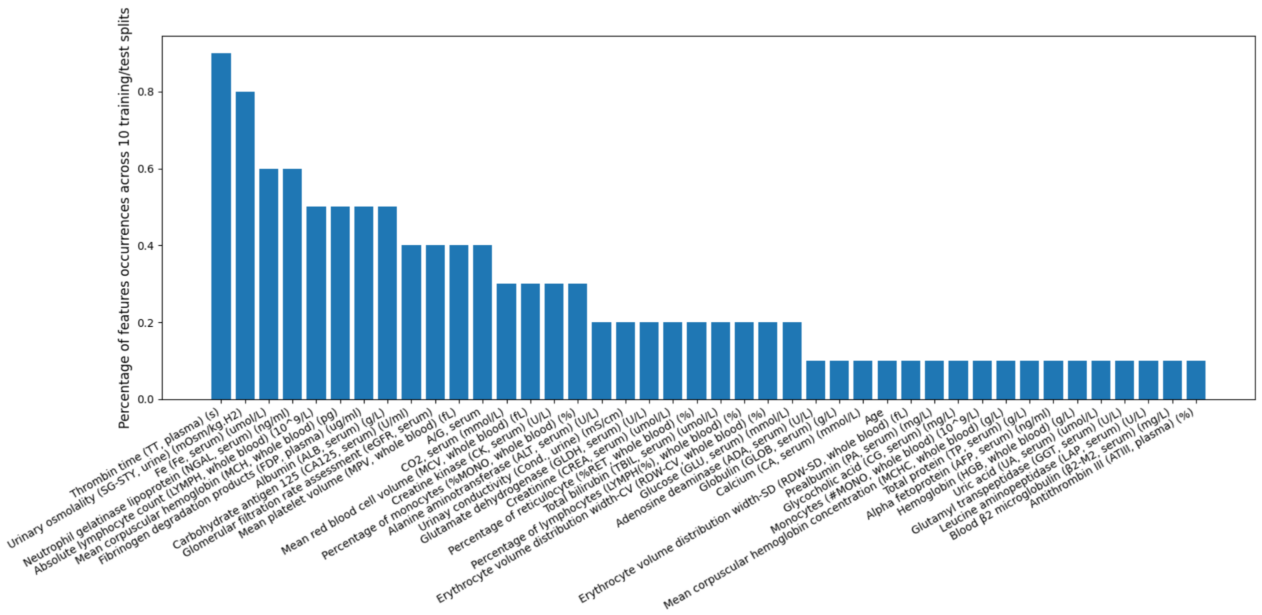

Supplementary Figure 2. The percentage of predictive feature occurrences across 10 different training/test spits.

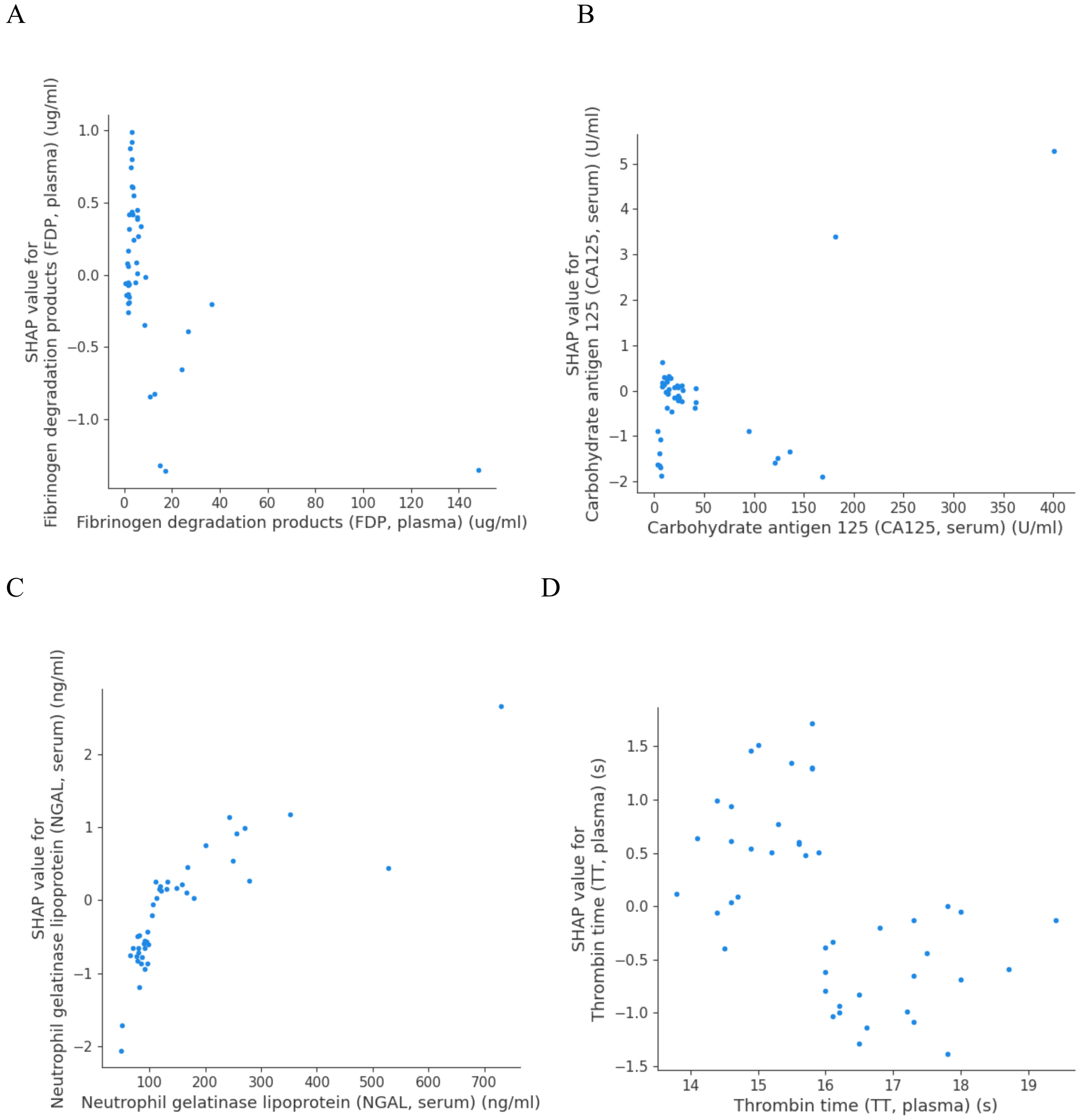
A
SHAP value for Fibrinogen degradation products (FDP, plasma) (ug/ml)
Fibrinogen degradation products (FDP, plasma) (ug/ml)
B
SHAP value for Carbohydrate antigen 125 (CA125, serum) (U/ml)
Carbohydrate antigen 125 (CA125, serum) (U/ml)
C
SHAP value for Neutrophil gelatinase lipoprotein (NGAL, serum) (ng/ml)
Neutrophil gelatinase lipoprotein (NGAL, serum) (ng/ml)
D
SHAP value for Thrombin time (TT, plasma) (s)
Thrombin time (TT, plasma) (s)

E

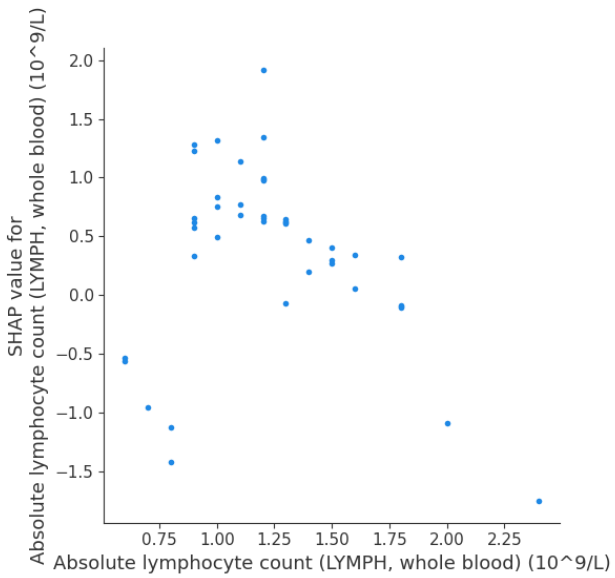

Supplementary Figure 3. The SHAP partial dependence plots of selected laboratory features for PFS prediction in LightGBM.
